# Effect of Flocculants Residue on Rheological Properties of Ultra-Fine Argillaceous Backfilling Slurry

**DOI:** 10.3390/ma15186485

**Published:** 2022-09-19

**Authors:** Shuai Li, Zheng Yu, Boyi Hu, Haoxuan Yu, Xinmin Wang

**Affiliations:** School of Resources and Safety Engineering, Central South University, Changsha 410083, China

**Keywords:** ultra-fine argillaceous backfilling slurry, rheological properties, flocculation residue, slurry pipeline transportation, flocculant network structure

## Abstract

Tailings concentration is indispensable for backfilling. Additionally, the residual flocculants in the concentration process affect the rheological properties of ultra-fine argillaceous backfilling slurry (e.g., viscosity and yield stress), resulting in a great effect on the fluidity and resistance of pipeline transportation. In this study, to explore the effect of flocculants residue on the rheological properties of the slurry, a series of rheological tests (constant shear rate test and variable shear rate test) were performed by changing the type, dosage, stirring time, temperature of flocculants addition and the amount of binder added. The results showed that the addition of flocculants increased the viscosity and yield stress of slurry. At a certain amount of flocculants additive, the flocculant network structure reached the best development state, which had a positive effect on increasing slurry viscosity and yield stress. As the stirring time increased, the scale of damage to the flocculant network structure became larger, which had a negative effect on increasing slurry viscosity and yield stress. Low temperature weakened the adsorption and bridging effect of polymeric chains, resulting in a poorly developed flocculant network structure, which had a negative effect on increasing slurry viscosity and yield stress. Caused by hydration products, the viscosity and yield stress of slurry with binder further increased. This study is significant for an in-depth study of the rheological and pipeline transport characteristics of ultra-fine argillaceous backfilling slurry, optimising the selection of flocculants for ultrafine particles, guiding backfill parameters and improving the reliability of pipeline transport.

## 1. Introduction

The grade of the ore generally declines as the shallow mineral resources become increasingly exhausted. The grinding grain size has become generally finer and finer in order to improve the beneficiation recovery rate, resulting in higher argillaceous composition and finer particle size [1,2,3,4,5]. Therefore, it is necessary to add a large number of flocculants to meet the concentration and sedimentation requirements of fine particles, which lead to many flocculants remaining in the concentrated tailings slurry. Flocculant is one of the commonly used agents in the field of wastewater treatment. Its principle is that the flocculant with a positive (negative) electric group and the water with a negative (positive) electric group makes it difficult to separate some particles or particles close to each other, reducing its potential so that it is in an unstable state, and the use of its polymeric nature makes these particles concentrated and separated by physical or chemical methods. With the general decrease in ore grade and the continuous progress of mineral processing technology, the grinding size becomes finer and finer, and the proportion of −200 mesh in metal mine tailings generally exceeds 80%, with an average particle size of around 50 μm. While the fine grinding size significantly increases the mineral recovery, it also makes the concentration and dewatering of the tailings more difficult. By relying on traditional natural settling, fine size tailings settle slowly, with low concentration efficiency and turbid overflow water. Flocculants must be added to accelerate the settling of fine particles and to ensure the stability and efficiency of the concentration process. Therefore, the purpose of adding flocculants to the mine filling process is to accelerate the settling rate of fine particle size tailings rather than to achieve efficient and low-resistance pipeline transport [6,7].

It was found that the residual flocculants combine with the fine particle size components in the slurry to form a stable flocculant network structure, which has a great effect on its rheological properties and pipeline transportation characteristics.

Many authors studied the influence of other factors on the rheological properties of backfill slurry: Sandeep et al. [8] evaluated the effect of hydration age, binder and superplasticiser dosages on the rheology of CPB; Cheng et al. [9] established a calculation model of paste resistance considering the effects of time and temperature and analysed the coupled effects of time and temperature on the rheological properties of CPB; Wu et al. [10] demonstrated the coupled effect of cement hydration and temperature on rheological properties of fresh cemented tailing backfill slurry. However, little research has been conducted on the effect of flocculants on the rheological properties of ultrafine-filled slurries [11,12,13]. For example, Xu et al. [14] presented the results of an experimental study on time-dependent rheological properties and mechanical performance of fresh cemented tailings backfill (CTB) mixtures with flocculants and only revealed the flocculants have a negative influence on rheological behaviour and mechanical strength of fresh CTB. Due to the complexity of the flocculation mechanism and flocculant network structure, most previous studies have simplified the ultrafine backfill slurry to a Bingham with time-independent flow characteristics and have derived and calculated the corresponding empirical equations for pipeline resistance without considering the effect of time.

Nevertheless, time-dependent rheological characteristics were observed in the pipeline transportation engineering example: the initial resistance is high, and it takes about 20~30 min to decrease to a stable value. Therefore, a comprehensive study of the effect of residual flocculants on the rheological characteristics of ultrafine backfill slurries and pipeline transport resistance is important to optimise the selection of flocculants for ultrafine particles, guide backfill parameters and improve the reliability of pipeline transport [15].

## 2. Experiment

### 2.1. Materials

**Figure 1:**
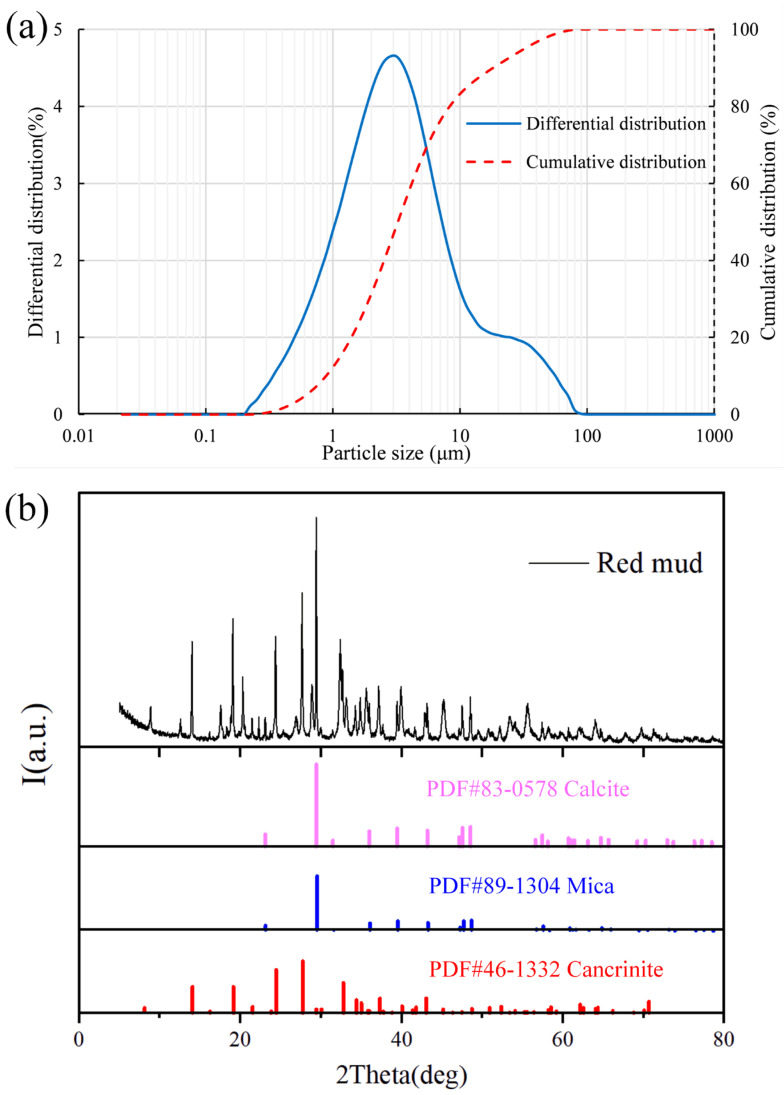
Physical and chemical properties of red mud: (**a**) particle size distribution of the red mud; (**b**) XRD of red mud.

Red mud is a representative ultrafine particle size solid waste. The red mud samples were dried in a drying oven set at 100 °C, and they were tested for particle size distribution, physical parameters and chemical composition later. The particle size distribution and the physical parameters of the red mud were analysed using a particle size analyser, and the test results are presented in Figure 1 and Table 1. As shown in Figure 1a, 66% of the particles are finer than 5 μm while only 4% of the particles are larger than 38 μm; as a result, red mud particles can be considered ultrafine size grained particles as ultra-fine particles [16]. As shown in Table 1, the coefficients of uniformity (Cu) and curvature (Cc) were 4.741 and 0.947, respectively. The hydraulic conductivity (K) was 3.35 × 10^−7^ cm/s. The specific surface area was 2940 m^2^/kg, which was much higher than that of ordinary Portland cement. The chemical compositions of red mud were analysed by X-ray diffraction (XRD) spectrometer, and the result are shown in Figure 1b. It indicated that the main chemical compositions of tailing were calcium alumina, hematite, Ca_3_AlFeSiO_4_(OH)_2_, alumina trihydrate, alumina monohydrate and calcite, and none of these mineral components had potential hydration properties.

Polyacrylamide-based flocculants are commonly used in the concentration process of ultra-fine tailings because of their low price and good effect. Polyacrylamide-based flocculants were selected as the flocculants in this experiment, including cationic-type polyacrylamide (ionic degree: 10, 30, 50), anionic-type polyacrylamide (molecular weight: 6 million, 8 million, 10 million, 12 million, 15 million) and nonionic-type polyacrylamide (molecular weight: 5 million, 10 million, 20 million).

In order to make the backfill body keep a certain strength, ordinary Portland cement (P.O32.5) is usually added to the slurry as a binder while preparing cemented paste backfill. P.O32.5 was selected as the binder in the experiment. Tap water from the test lab was used for the test.

### 2.2. Experimental Methods

**Figure 2:**
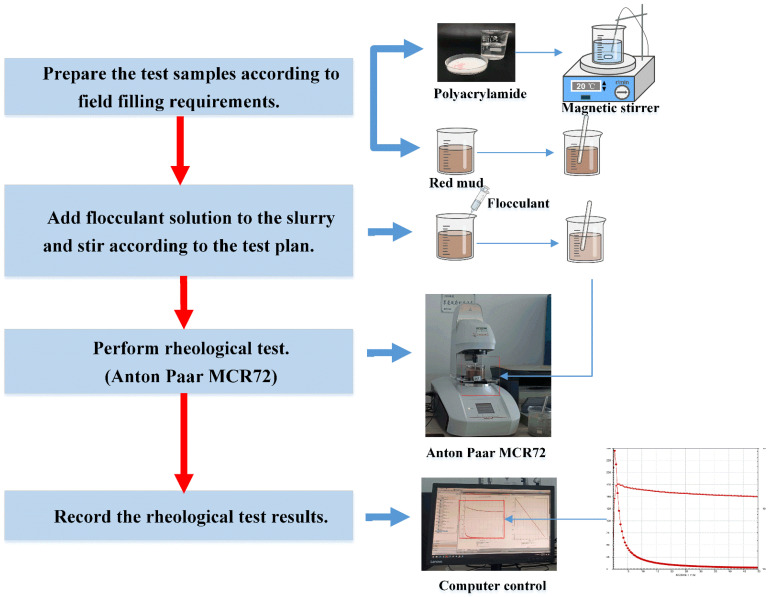
Experimental flow chart.

As shown in Figure 2, the test process mainly includes the preparation of flocculants and slurry, the preparation of test samples according to the test plan and a rheological test.

#### 2.2.1. Specimen Preparation

We added 0.5 g flocculants into a beaker containing 500 mL of water, and the mixture was stirred for more than 1 h to obtain a fully dissolved flocculants solution using a magnetic stirrer. (Flocculants concentration was 1‰, and 2.5 mL flocculants solution dosage was equivalent to 5 g/t.) Then, the slurry with a mass concentration of 60% was prepared, which was the most suitable for the actual requirements of mine according to the filling ratio test conducted indoors. Finally, fully diluted and dissolved polymer flocculants polyacrylamide with different ionic types (cationic, anionic and nonionic) and molecular weights (6~20 million) were added to the prepared slurry, respectively.

The polymer flocculants had to be sufficiently dissolved and activated by agitation to be more conducive to flocculation and settling reactions with the fine-grain tailings. With all other conditions being equal, the addition of APAM to the ultrafine size tailings more than doubled the settling rate at the solid–liquid separation surface compared to PAM and CPAM, resulting in a denser and larger floc, floc and floc network structure. The same results were obtained in the dynamic and static flocculation and sedimentation tests. The reason for this is that the anionic flocculant has a more pronounced flocculation bridging and adsorption effect with the ultra-fine red mud, resulting in a denser and larger floc, floc and floc net structure, which in turn results in a faster settling rate and a higher clarification of the overflow water. For this reason, APAM is often used to accelerate the settling of fine-grained tailings in the actual mine filling process, with special flocculant dissolution and preparation units.

#### 2.2.2. Rheological Tests

In this test, Anton Paar MCR72 modular intelligent advanced rheometer was selected to measure the rheological parameters of slurry. The rheological tests were carried out under the conditions of constant shear rate and variable shear rate.

**Figure 3:**
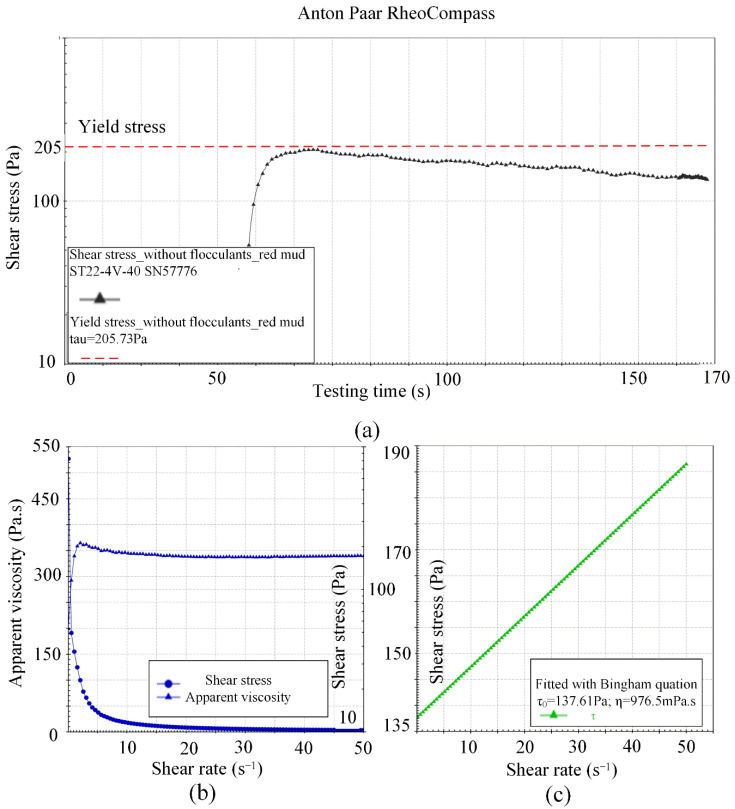
Flow curves of CPB under different shear conditions: (**a**) shear time vs. shear stress at constant shear rate; (**b**) shear rate vs. shear stress and apparent viscosity; (**c**) shear rate vs. shear stress fitted with Bingham equation.

During the constant shear rate (0.3 s^−1^) rheological test, the rheometer recorded the change in shear stress with time. As shown in Figure 3a, the shear stress increased to the peak with shear time and then decreased with time, and the peak shear stress value of slurry was taken as the yield stress value.

During the variable shear rate rheological test, the changes in shear stress and apparent viscosity of slurry with shear rate were automatically recorded by rheometer. In the test and analysis process of shear stress changing with shear rate, the total test time was 180 s, and a total of 100 sets of data were obtained; that is, a set of shear stress was tested every 1.8 s, and the corresponding change rate of shear rate was 0.5/1.8 s^−2^. As shown in Figure 3b, the shear stress fluctuated at first with the increase in the shear rate, then increased approximately linearly with the shear rate, and the apparent viscosity decreased exponentially with the shear rate. As shown in Figure 3c, shear stress variation with the shear rate was fitted with Bingham equation by rheometer. The Bingham equation is as follows:

(1) $$\tau =\tau_{0}+\eta \frac{du}{dy}$$

where *τ_0_* (Pa) is the yield stress; *η* (Pa·s) is the stiffness coefficient or plastic viscosity coefficient [17].

## 3. Results and Discussion

### 3.1. The Rheological Behaviour of Red Mud with Different Flocculants

**Figure 4:**
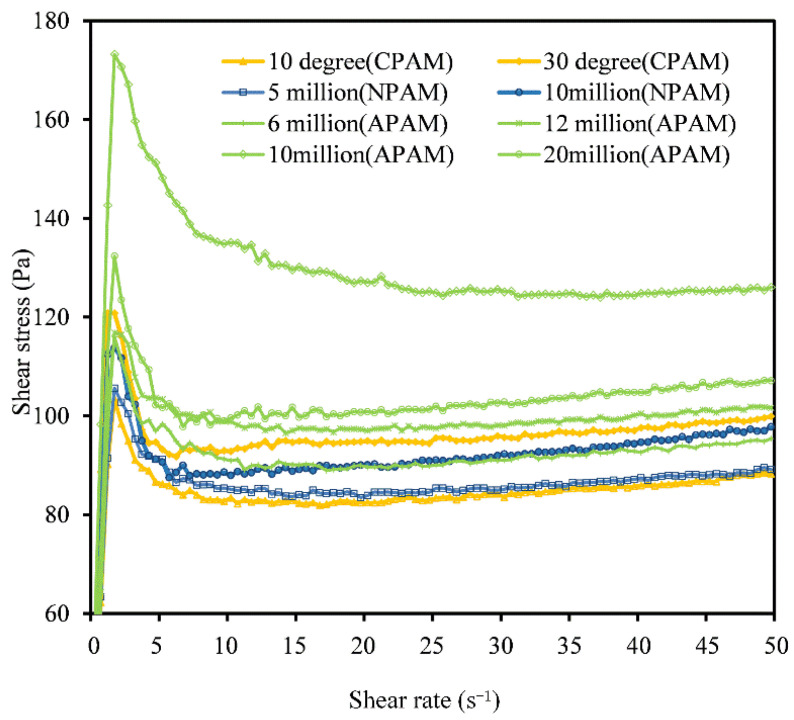
Flow curves of red mud with different ionic types: shear rate vs. shear stress.

In the rheological test, three types of flocculants, including 12 models, were separately mixed into red mud samples. The change in shear stress with shear rate during the test was recorded by the rheometer, and the shear stress vs. shear rate curves of red mud with different types of flocculants are shown in Figure 4.

Figure 4 shows the distribution range of shear stress vs. shear rate curves of red mud after adding different types of flocculants.

The shear stress of the samples with APAM varied from 90 Pa to 160 Pa; as a result, their curves were at the top. The shear stress of the samples with CPAM and with NPAM varied from 80 Pa to 100 Pa; their curves were at the bottom. It can be explained that APAM was more effective in flocculation bridging and adsorption coagulation with ultrafine red mud. Therefore, the flocs and flocculant networks formed by APAM had a denser structure and larger volume, which made the measured shear stress value higher [18].

Flocculants of the same ionic type can be further subdivided into different types according to molecular weight or ionic degree.

**Figure 5:**
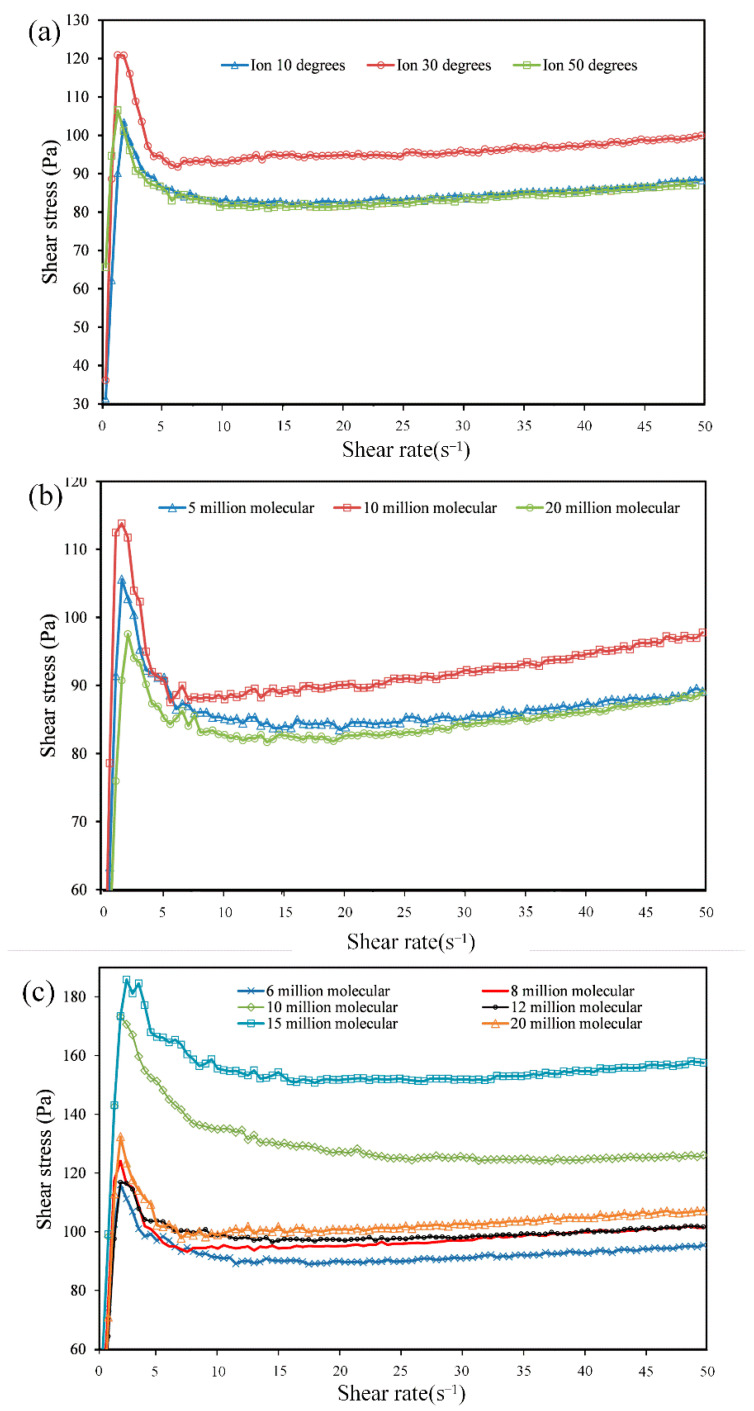
Flow curves of red mud with different ionic types: (**a**) cationic flocculants with different ionic degrees; (**b**) nonionic flocculants with different molecular weights; (**c**) anionic flocculants with different molecular weights.

Figure 5 shows the flow curves of slurry with three ionic types of flocculants of different molecular weights or different ionic degrees. It can be seen from Figure 5c that the measured shear stress increased with the increasing molecular weight of anionic flocculants, but the shear stress value decreased rapidly as the molecular weight increased to 20 million. It can be explained that increasing the molecular weight of anionic flocculants in an appropriate range was beneficial to improve the flocculation sedimentation effect of ultrafine size red mud, while the excessive molecular weight of flocculants would have a negative effect on its flocculation. As shown in Figure 5a,b, cationic flocculants and nonionic flocculants had similar rules.

Under the same test conditions, the higher the shear stress of a sample means that the flocculants added to the sample have a better flocculation effect. When comparing the shear stress vs. shear rate curves of the three types of flocculants, the samples with the addition of anionic flocculants generally had higher shear stress, which means that the anionic flocculants had the best flocculation effect and were most suitable for red mud paste-like. Therefore, the subsequent tests mainly focused on anionic flocculants, exploring the effect of flocculants on the rheological properties of the ultra-fine slurry.

**Figure 6:**
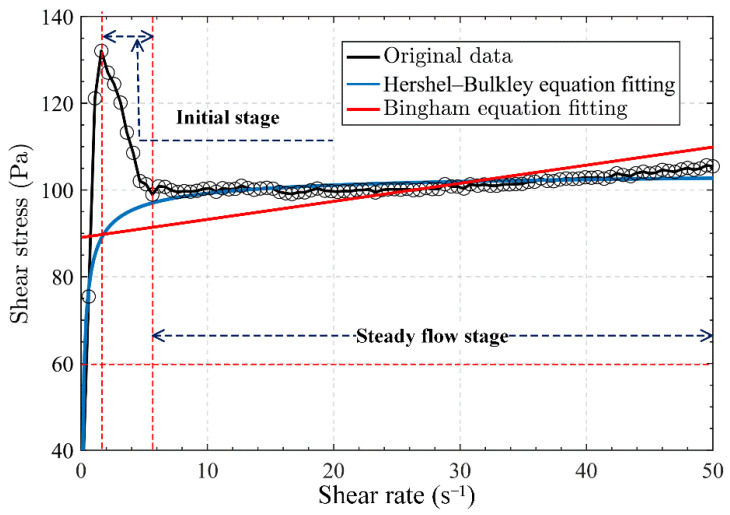
Fitting flow curve comparison.

As shown in Figure 6, the shear stress vs. shear rate curve can be divided into three phases. At the initial phase of the experiment, shear stress surged to the maximum value, with the shear rate increasing to about 2.0/s. The main reason for this behaviour is that the slurry was stationary while its internal flocculent network structure was developed at the initial phase; consequently, large shear stress is needed to transform from a static state to a rheological state. Then, the shear stress decreases slowly as the slurry transforms from a static state to a rheological state. In the end, the slurry turned to a stable flow state; consequently, shear stress increased linearly, with the shear rate increasing to about 10/s.

The shear stress vs. shear rate curve was fitted with the Bingham equation to analyse the rheological properties of slurry by the rheometer, but the data in the initial stage of the test fluctuated so that the fitting result deviated greatly from the actual situation. The rheological properties of slurry were mainly measured by the change in shear stress in its steady flow stage rather than the initial stage. Additionally, the curve was refitted well with the Hershel–Bulkley equation, and the fitting degree of the curve was 98%. Thus, the data before slurry flow and at the beginning of the flow state are supposed to be eliminated and refitted with the Hershel–Bulkley equation to reflect the variation in shear stress with a shear rate more accurately. The Hershel–Bulkley equation is as follows:

(2) $$\tau =\tau_{0}+\mu {\dot{\gamma }}^{n}$$

where *τ* (Pa) is the shear stress; *γ* (1/s) is the shear rate and *n* is the Hershel–Bulkley (H-B) index; and *τ_0_* (Pa) and *μ* (Pa·s) are the initial yield shear stress and plastic viscosity, respectively [9].

### 3.2. Effect of Ratio of APAM Additive

In order to accelerate the dewatering of the tailings in the project, it is necessary to determine a suitable flocculant addition ratio. In the experiment of exploring the effect of the ratio of APAM additive on fluidity, five test samples were prepared for rheological experiments, among which four samples were added with 15 million molecular weight flocculants of 0 g/t, 5 g/t, 10 g/t, 15 g/t and 20 g/t, respectively, while the other group as the control group without adding APAM (0 g/t).

**Figure 7:**
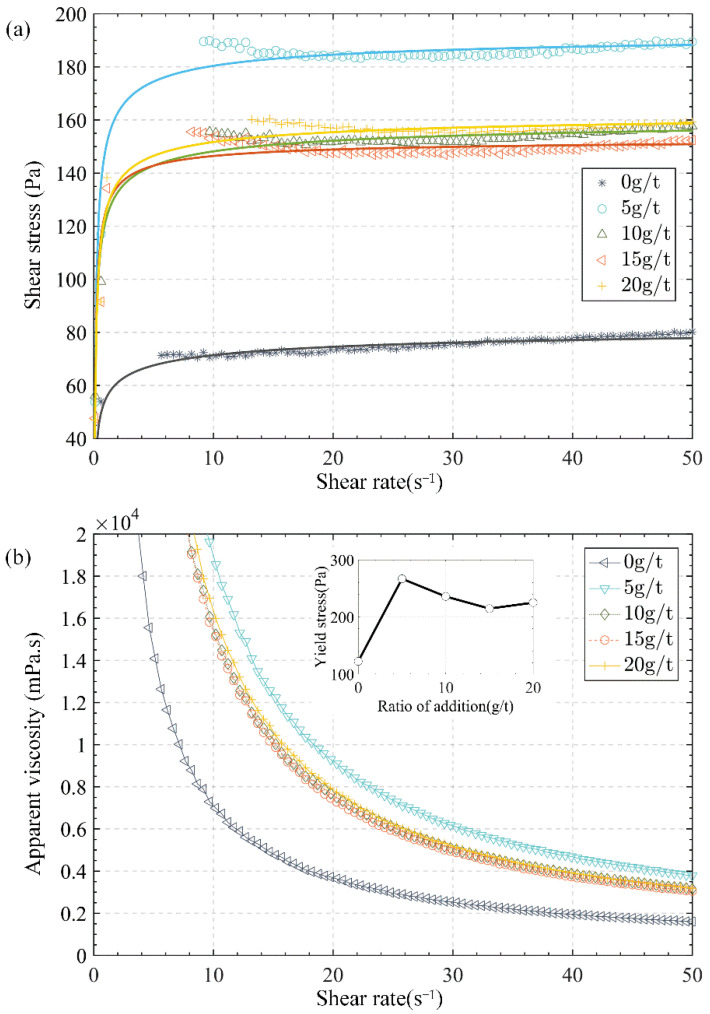
Flow curves of samples with various APAM addition: (**a**) shear rate vs. shear stress; (**b**) shear rate vs. apparent viscosity. The yield stress was presented as a function of ratio addition of APAM in the inset.

Figure 7 shows the shear stress and apparent viscosity of different ratios of APAM additive. As shown, the shear stress in the steady flow stage increased approximately linearly with respect to the shear rate, while the apparent viscosity decreased exponentially. In addition, the changing trends of these two rheological parameters were highly consistent, so only yield stress and shear stress were analysed to measure the rheological properties of the slurry. When comparing the flow curves of samples with different ratios of APAM additive, the yield stress increased from 123 to 267 Pa (maximum value), with the ratio of APAM additive increasing from 0 to 5 g/t, while it was in a downward trend with the ratio of APAM additive over 5 g/t.

As shown in Figure 7a, the shear stress of the samples with APAM increased significantly compared with the sample without APAM. Moreover, with the addition ratio of 5 g/t APAM as the cut-off point, the shear stress increased with the ratio of APAM additive increasing from 0 to 5 g/t, and the shear stress curves moved down and converged when the addition ratio of APAM was more than 5 g/t, concentrated in the region, which was consistent with the change law of yield stress. According to the research results of Wang et al. [19], the flocculant network structure formed by APAM was stronger than the flocculant network structure formed by particle self-flocculation, which corresponded to the test results. It is also known from the test results that excessive flocculants had negative effects on the flocculant network structure.

### 3.3. Effect of Stirring Time

In practice, the slurry would be stirred by the deep cone thickener to ensure the flocculants react fully, which may affect the action of the flocculants. In the experiment exploring the influence of stirring time on the APAM, four test samples with 10 million molecular weight flocculants were prepared, and the samples were stirred for 3 min, 6 min, 9 min and 12 min, respectively, under the same other conditions.

**Figure 8:**
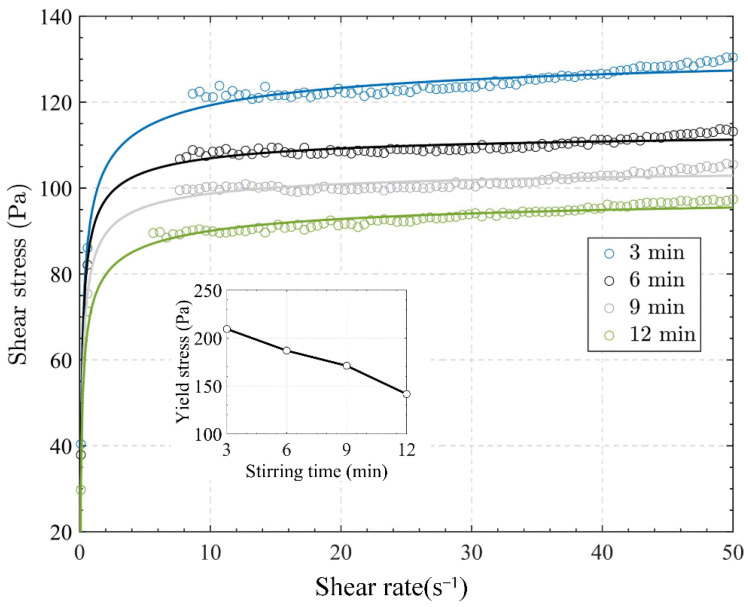
Flow curves of samples with different stirring times: shear rate vs. shear stress and the yield stress were presented as a function of stirring time in the inset.

Figure 8 shows the shear and yield stresses for slurries stirred at different times, respectively. As shown, the shear stress of slurry decreased with the stirring time increasing, and the yield stress decreased from 210 to 142 Pa with the stirring time increasing from 3 to 12 min.

The effect of stirring time on rheological properties can be explained as follows: The stirring in a laboratory test or deep cone thickener is essentially a shearing action, which would destroy the floc structure. Moreover, as the specimens were stirred for longer, the continuous shear damage would be stronger, which made the shear stress more reduced.

### 3.4. Effect of Temperature

The environment temperature of mines is variable; for example, the temperature difference between winter and summer in northern China reaches 30~40 °C, and the effect of flocculants may differ at different temperatures.

In the experiment to explore the influence of temperature on the APAM, two samples with 20 million molecular weight APAM were prepared, exploring the changes in filling slurry fluidity at 5 °C and 20 °C under the same other conditions.

**Figure 9:**
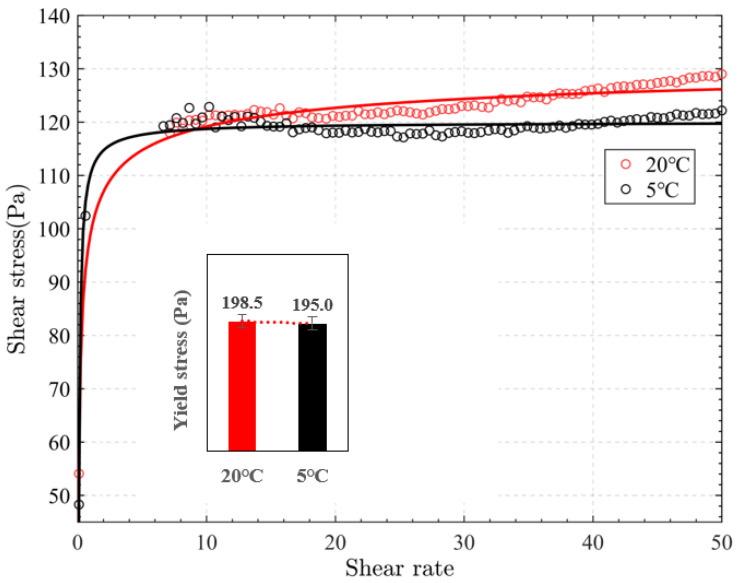
Flow curves of samples with different temperature: shear rate vs. shear stress and the yield stress was presented as a function of stirring time in the inset.

Figure 9 shows the effect of temperature on the shear stress and the yield stress of slurry. As shown, the yield stress decreased from 198.5 to 195.0 Pa, with the stirring time increasing from 3 to 12 min. It is evident that the shear stress of slurry decreased with the temperature dropping from 20 °C to 5 °C (i.e., when the temperature was low, the flocculation effect of APAM was not ideal).

According to the research results of Wang et al. [20], low temperatures would limit the dissociation of flocculant and result in the loss of the bridging effect, which corresponded to the test results. Overall, the influence of temperature on APAM existed, but the influence was relatively low.

### 3.5. Effect of C/T Ratio

It is necessary to add a certain proportion of cementing material into the slurry in order to make the backfill body have the strength to support the goaf.

**Figure 10:**
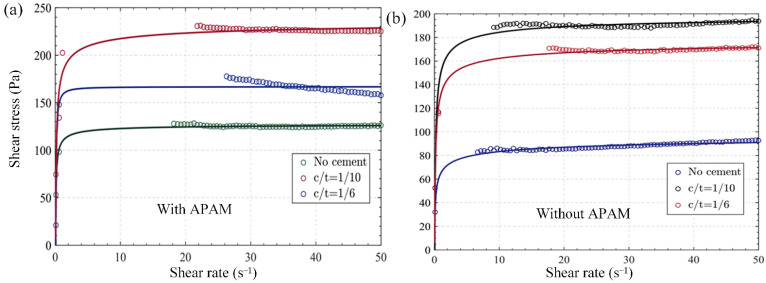
Flow curves of samples with different C/T ratio: (**a**) shear rate vs. shear stress (without APAM); (**b**) shear rate vs. shear stress (with APAM).

Figure 10 shows the effect of the c/t ratio on the shear stress of slurry without and with APAM, respectively.

It can be observed from both Figure 10a,b that the shear stress was higher for slurry with cement added than for slurry without cement added, which is most likely caused by more hydration products in specimens with cement added. Moreover, under the same condition of c/t ratio, the shear stress was also higher for slurry with APAM than for slurry without APAM. Above all, adding flocculants and cement would make the slurry difficult to shear and make its fluidity worse. Moreover, the negative effects of the two on fluidity did not conflict, which can make the slurry more difficult to shear [21]. According to the research results of Jiang et al. [1], the zeta potential of the cement particles and hydration products decreased because of the addition of APAM, leading to the weakening of repulsive force and subsequently showing worse flow behavior, which corresponded to the test results.

### 3.6. The Effect of Residual Flocculants in Slurry Transportation

The slow settling speed of ultra-fine tailings, low concentration efficiency and high turbidity of the overflow water necessitated the addition of flocculants to accelerate the settling of fine particles, which can therefore be left in the concentrated paste-like body, affecting its rheological and pipeline characteristics. The flocculant residue led to increased viscosity, low concentration, easy caking and increased resistance to pipeline transport, resulting in increased wear and tear on the pipeline, reduced flow rate at the outlet and shorter distance for self-flow transport by the slurry’s own weight.

In the case of the Shizhudi gold mine in Songxian, Henan Province, for example, a large amount of flocculant remained in the paste-like body of the ultra-fine tailings, leading to an increase in viscosity and a more prominent agglomeration phenomenon during dewatering in the ceramic filter and belt conveying, reaching a paste-like state at a mass concentration of 58% to 60%. Due to the high viscosity of the tailings, the risk of blocking the pipe was extremely high in the case of a filling multiplier of only four (under the condition of 70 m vertical borehole length and approximately 200 m horizontal pipeline length) in the first filling goaf area of the mine, resulting in an outlet flow rate of less than 2 m/s for the filling slurry.

## 4. Mechanism Analysis

Compared with the samples without flocculants, adding flocculants would have a negative impact on the fluidity of slurry, and its mechanism analysis mainly started from two aspects, including flocculation bridging and water migration mechanism. Organic flocculants would hydrolyse into long-chain polymers after being dissolved in water. The internal water structure of slurry greatly affected its fluidity during pipeline transportation. The types of water mainly included free water, bound water, viscous water and floc water, among which free water had the best fluidity and was the main carrier of pipeline transportation. Based on the double electron layer structure of fine tailings particles, the water molecules in the adsorption layer were closely arranged on the surface of tailings particles to form bound water under the action of static electricity, while the adhesion of water molecules in the diffusion layer was slightly weak to form viscous water, and the water in the two layers was collectively referred to as adsorbed water. Moreover, the flocculant network structure formed by flocculant molecular bridging would wrap the free water in the slurry, which would be transformed into flocculent water with poor fluidity, which would make the fluidity of the slurry worse [19].

**Figure 11:**
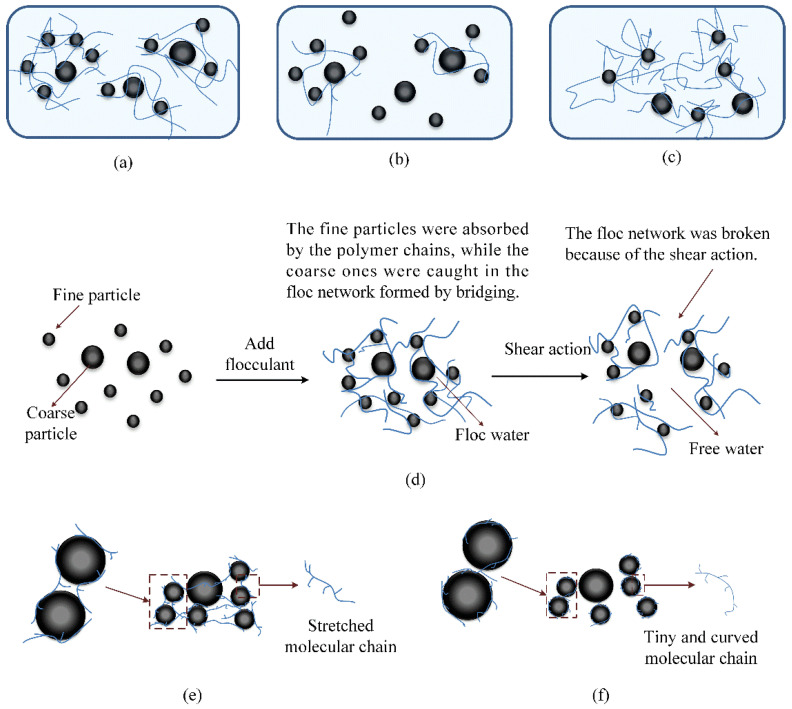
Flocculant network structures under different conditions: (**a**) low additive ratio; (**b**) reasonable additive ratio; (**c**) excessive additive ratio; (**d**) shear action; (**e**) warm temperature (20 °C); (**f**) low temperature (5 °C).

The polymer molecules in the flocculant captured the fine particles in the slurry, absorbed the vacancies on the particles, and the polymer chains built bridges for the particles to form a flocculant network structure, making it more difficult for the tailings particles to slide against each other during the shearing process, ultimately increasing the slurry yield stress. There was a negative charge on the surface of fine tailings particles, and a repulsive force existed between particles. Polyacrylamide used in the experiment was an organic polymer flocculant. The charge generated by its ionisation would reduce the potential of fine-sized tailings and compress the double electron layers, which would weaken the repulsive force between tailings particles, increase the friction and collision between particles and enhance the adsorption bridging effect. Figure 11 shows flocculant network structures under different conditions [3,22,23,24].

Firstly, the addition of flocculants would affect the fluidity of the slurry; moreover, the dosage of flocculants added would make the adsorption bridging mechanism different, which would eventually lead to different effects of flocculants on the fluidity of the slurry. Generally, the ratio of APAM additive can be divided into low additive ratio, reasonable additive ratio and excessive addition ratio:

- As shown in Figure 11a, the dosage of flocculants was less at a low additive ratio, and there were still some free fine particles in the slurry that were not captured by polymer molecules or captured particles with adsorbed vacancies. Because there were few polymer molecules objectively, the particles bridged by polymer molecular chains were also very limited, resulting in loose floc structure and less floc water formed. With the increase in flocculants addition, as shown in Figure 11b, all the fine particles in the slurry could be adsorbed completely, and there were no extra adsorption vacancies on the particles at the reasonable additive ratio. At this time, the polymer molecular chain could bridge the fine particles in the slurry to the greatest extent, and the formed floc structure was the most stable, which could wrap more water. If the dosage of flocculants continued to increase, as shown in Figure 11c, there would be no free fine particles in the slurry. The polymer molecules tended to adsorb the remaining vacancies of the originally attached particles, and only a few polymer molecules adsorbed vacancies on other particles outward. As a result, the bridging effect of polymer chains was weakened, resulting in a relatively loose flocculant network structure and less floc water formed by wrapping [15,25];
- Secondly, the flocculant network structure formed by bridging was only a relatively stable structure, which can be destroyed by shear reaction (continuous stirring). Under the continuous action of external shear force, as shown in Figure 11d, the floc structure was split into small flocs due to the fracture of long-chain molecules, and the fine tailings particles originally agglomerated together were dispersed to many small flocs. Furthermore, a large number of water molecules originally adsorbed and contained were also released. In addition, the degree of damage to the floc structure would be deepened with the increase in stirring time [26,27];
- Thirdly, temperature could affect the adsorption bridging effect of flocculants. Under a suitable temperature condition, the flocculants were fully dissociated, and the polymer molecular chain was extended, which met the requirements of chain adsorption particles. As shown in Figure 11f, the dissociation of flocculants was limited at a lower temperature (5 °C), and the molecular chains of flocculants became tiny and curved, which promoted the adhesion of polymer molecules on the surface of particles. The polymer molecules tended to adsorb the remaining vacancies of the originally attached particles instead of outward adsorbing the vacancies on the surrounding particles, thus inhibiting the adsorption bridging effect of flocculants molecules [27,28].

Additionally, the free water in the slurry was consumed by cement and thus converted into bound water. The cohesive force and interparticle frictional resistance increased with hydration (e.g., calcium silica hydration gel, ettringite, calcium hydroxide), resulting in a decrease in fluidity. In addition, floc structure and hydration products increased the friction and adhesion between particles, which made it difficult for particles to slide each other during shearing [29,30,31,32].

## 5. Conclusions

The following conclusions can be drawn based on the results:

1. The addition of flocculants increased the viscosity and yield stress of slurry. The shear stress of the samples with APAM varied from 90 Pa to 160 Pa, which was much higher than NPAM and CPAM. Additionally, by combining the settling rate performance of the three flocculants, it can be concluded that APAM was the best flocculant for selecting ultra-fine particles;
2. At a certain amount of flocculants additive, the flocculant network structure reached the best development state, which made yield stress 118% higher than slurry without APAM and 18% higher than with excessive APAM. It is suggested that the rheological and sedimentation tests should be conducted in advance to explore the best addition of flocculant in order to obtain the best flocculation effect;
3. Prolonging the stirring time and reducing ambient temperature can weaken the adsorption and bridging effect of polymeric chains, and long shear time increases the damage to the flocculant network structure. The yield stress decreased from 210 to 142 Pa, with the stirring time increasing from 3 to 12 min. In the lower temperature environment, the flocculation effect of flocculants was poor due to molecular bending. The effect of temperature was less, and low temperature only reduced the yield stress by 2%. It is theoretically feasible to reduce the negative effects of residual flocculants in pipeline transportation by prolonging stirring time and lowering temperature;
4. A large number of hydration products were generated during the hydration reaction (e.g., calcium silica hydration gel, ettringite, calcium hydroxide), which increased the friction and adhesion between particles. When comparing the flow curves with and without cement, the shear stress of the slurry, which was added with cement, increased significantly. Additionally, the shear stress of slurry with APAM action was further increased, in which the shear stress of slurry of 1:10 c/t ratio was increased by 44%. In summary, both flocculants and cement increase the resistance of filling slurry pipeline transportation, which adversely affects pipeline transportation.

## Figures and Tables

**Table 1:** Physical parameters of the red mud.

Density (g/cm^3^)	K (cm/s)	Median Grain Size (μm)	Specific Surface Area (m²/kg)	Cu	Cc	pH
2.424	3.35 × 10^−7^	3.248	2940	4.741	0.946	12.1

## Data Availability

Not applicable.
